# Understanding the Reasons for Receiving HPV Vaccination among Eligible Adults in Italy

**DOI:** 10.3390/vaccines12070728

**Published:** 2024-06-29

**Authors:** Grazia Miraglia del Giudice, Vincenza Sansone, Giorgia Della Polla, Italo Francesco Angelillo

**Affiliations:** Department of Experimental Medicine, University of Campania “Luigi Vanvitelli”, via Luciano Armanni 5, 80138 Naples, Italy

**Keywords:** HPV, Italy, reasons, survey, uptake, vaccination

## Abstract

Background: This cross-sectional survey aimed to explore the reasons for receiving the HPV vaccination among eligible adults in Italy. Methods: The survey was conducted from July 2023 to April 2024 in Naples, Southern Italy. Results: A total of 282 questionnaires were collected. The majority of the respondents (73.2%) was aware that HPV vaccination was recommended and this was more likely among women, healthcare workers (HCWs) or students in health sciences, and those who had acquired information from physicians. The most frequently cited reasons for vaccinating were self-protection from the infection (77.6%) and from cervical/oral/penile/anal cancer (68.9%), knowing that the vaccination was free of charge (46.2%), awareness of the severity of the HPV disease (43%), to protect their partner (42.6%), and perception of being at risk (24.2%). Being HCWs or students in health sciences, believing that HPV infection could cause a serious disease, and having a higher number of oral intercourse experiences in the last year were significant predictors of the perception of being at risk. Female and Italian respondents were more likely to receive the HPV vaccination because it was effective in preventing cancer. Conclusions: Targeted educational programs and health interventions should be developed to ensure enhancing knowledge and fostering positive attitudes toward the HPV vaccination.

## 1. Introduction

In Italy, almost 5000 newly diagnosed cancer cases are attributed to chronic infections of oncogenic strains of human papillomavirus (HPV) each year [1]. In order to effectively prevent cervical and other HPV-related cancers, the HPV vaccination is one of the most important public health measures. In Italy, it has been included in the national immunization schedule and offered free of charge to adolescent girls aged 12 years since 2008, with the goal of achieving 95% coverage in 5 years [2], and to boys aged 12 years since 2018. Presently, according to the National Immunization Program, it is also recommended as a catch-up vaccination until the age of 26 and 18 for women and men, respectively. Moreover, some regions offer the vaccination free of charge to women who have been treated for cervical intraepithelial neoplasia grade 2 (CIN2+) or higher-grade lesions, subjects with HIV, and men who have sex with men [3]. However, despite the ample evidence regarding the effectiveness of this vaccination and its recommendation, individuals continue to underestimate the risk of acquiring the infection, and it is of major concern that completion of the vaccine series remains significantly behind the target of 95%, with the highest coverage of 71.27% in girls (birth cohort 2005) and of 56.93% in males (birth cohort 2007) as of 31 December 2022 [4].

At present, the availability of data on the reasons for receiving HPV vaccination among eligible adults in various countries is limited [5,6,7]. This understanding is crucial to shed light on vaccine acceptance, and knowledge of these reasons will assist HCWs, public health authorities, and healthcare decision-makers in building targeted and relevant interventions to increase immunization coverages. With this in mind, it was of utmost importance to conduct this cross-sectional survey that aimed to explore the reasons for receiving the HPV vaccination and to determine the factors influencing such behavior among an adult eligible population in Italy.

## 2. Materials and Methods

### 2.1. Setting and Sample

The survey was conducted from July 2023 to April 2024 in a vaccination center of a Teaching Hospital situated in the city of Naples, Southern Italy. Faculty, staff, and students aged ≥18 years were invited via an intranet announcement to participate, if eligible, in a free-of-charge HPV vaccination campaign. Adult unvaccinated people eligible to receive this vaccination free of charge were defined as (i) individuals up to 26 years of age; (ii) women up to age 65 years who have been treated for cervical intraepithelial neoplasia grade 2 (CIN2+) or more severe lesions (before treatment or thereafter up to a maximum of three years after treatment); (iii) individuals with HIV; or (iv) men who have sex with men. A total of 283 individuals indicated that they were eligible, and all were invited to attend the vaccination center.

### 2.2. Data Collection

All attendees were approached by the members of the research team in the center’s waiting room and were informed of the purpose and methodology of the survey, the voluntary nature of participation, the anonymity and confidentiality of the information at all stages, that the data collected would be used only for research purposes, and that they were free to conclude their participation at any time without explanation. Those who provided verbal informed consent for their participation were asked to answer a self-administered questionnaire and to return it to the research team in a sealed envelope to ensure confidentiality. The participants did not receive any kind of compensation or incentive.

### 2.3. Questionnaire

The questionnaire was developed, in Italian and in English, from previous surveys conducted by some of us on vaccination attitudes and practices [8,9,10]. The feasibility, acceptability, and understanding of the questionnaire were determined before the formal distribution with 10 individuals who were included in the survey.

The questionnaire was divided into four distinct sections and the average time required for completion was approximately 10 min. At the beginning of the questionnaire, a set of inquiries concerning socio-demographic and health-related data were collected, comprising gender, age, partnership status, nationality, level of education, employment, sexual orientation, parents’ professional status, having chronic medical conditions, having family members with HPV-related cancer diagnosis, and having performed an HPV screening test. The second section was dedicated to the evaluation of the participants’ attitudes toward HPV infection and the associated vaccination using a total of 5 items, which gauged their belief if the HPV infection could cause serious disease and of the risk of acquiring HPV, their perception of the vaccination’s efficacy to prevent cervical/oral/penile/anal cancer, and their perception of the importance of the vaccination for women and for men. Responses for each of the questions were rated on a 10-point Likert-type scale, with scores of 1 indicating “not at all” and scores of 10 denoting “at all”. Respondents were also asked to state the reason/s for not having received the vaccination and for accepting it with the possibility of a multiple-choice answer. The third section regarded participants’ sexual behaviors. A question investigated whether they had ever had oral intercourse, with two answer options, “yes” and “no”. If the response was positive, three open-ended questions explored the age of first oral intercourse experience, the total number of partners, and the number of partners in the last year. A question investigated whether the participants had ever had complete sexual intercourse, with two answer options, “yes” and “no”. If the response was positive, three open-ended questions explored the age of the first experience of intercourse, the total number of partners, and the number of partners in the last year. Among those who had sexual intercourse, condom frequency with a five-point response option from “always” to “never” was asked about. Information about whether the participants were asked by/asked their partners about HPV vaccination status was requested with two questions with “yes” and “no” answer options. Among those who asked the partner, a question was used to evaluate whether the partner was vaccinated, with three answer options, “yes”, “no”, and “do not know”. Moreover, the willingness to suggest HPV vaccination to their partner was investigated with three answer options, “yes”, “no”, and “do not know”. In the last section, participants were asked whether they had acquired information about HPV and vaccination before this survey. Those who selected “yes” were asked to recall their source(s) of information, from a list of eight. All participants were asked whether they needed to receive information.

### 2.4. Statistical Analysis

STATA software version 18 was used to analyze the data. In the first step, a description of the characteristics of the sample was performed using frequencies and proportions for categorical variables and means, ranges, and standard deviations for continuous variables. In the second step, the chi-square test and Student’s *t*-test were used to estimate the predictors of the different outcomes of interest. In the third step, the independent variables with a *p*-value ≤ 0.25 in the univariate analysis were selected in the three multivariate logistic regression models to detect which were associated with the following outcomes: awareness that HPV vaccination was recommended for them before being informed by the vaccination center (no = 0; yes = 1) (Model 1); having received the HPV vaccination because they perceived themselves at risk of being infected (no = 0; yes = 1) (Model 2); and having received the HPV vaccination because they believed that it was effective in preventing cancer (no = 0; yes = 1) (Model 3). The following independent variables were tested in all models because they were potentially related to the dependent variables: age in years (continuous), gender (male = 0; female = 1), partnership status (married/cohabited with a partner = 0; unmarried/separated/divorced = 1), Italian nationality (no = 0; yes = 1), sexual orientation (bisexual/homosexual/pansexual = 0; heterosexual = 1), being an HCW or student in health sciences (no = 0; yes = 1), having at least one chronic medical disease (no = 0; yes = 1), having at least one HCW parent (no = 0; yes = 1), having acquired information regarding HPV vaccination from physicians (no = 0; yes = 1), and a need for additional information regarding HPV vaccination (no = 0; yes = 1). The following independent variables were tested for Models 1 and 2: believing that HPV infection could cause serious disease (continuous), believing that HPV vaccination was effective in preventing cancer (continuous), being aware that the HPV vaccination was recommended for them before being informed by the vaccination center (no = 0; yes = 1), having ever had complete or oral sexual intercourse experience (no = 0; yes = 1), the total number of oral sexual partners (continuous), the total number of complete sexual partners (continuous), the number of oral sexual partners in the last year (continuous), and the number of complete sexual partners in the last year (continuous). The independent variable of perception of the risk of being infected by HPV (continuous) was also added in Model 2. The values for variables’ entry and removal in the final models were, respectively, *p* = 0.2 and *p* = 0.4 throughout the stepwise selection procedure. The odds ratios (ORs) and their 95% confidence intervals (CIs) were calculated to assess the strength and direction of associations between independent variables and outcomes of interest. A two-sided *p*-value ≤ 0.05 was considered statistically significant.

## 3. Results

Of the 283 subjects approached, a total of 282 participated in the survey, with a response rate of 99.6%. The principal characteristics of the participants are presented in Table 1. The average age was 26.6 years, they were equally distributed by gender, a substantial majority were heterosexual (70.4%), 16.3% were not Italian, 20.6% of the women reported having had an HPV screening test and half of them tested positive, the vast majority had had complete sexual or oral intercourse and the mean age at the first intercourse experience was, respectively, 18.3 years and 17.6 years, condoms were often/always used during sexual intercourse by almost two-thirds, and the mean number of sexual partners in the respondents’ lifetime and in the last year were, respectively, 4 and 1.2.

The participants’ attitudes toward HPV and its vaccination, measured on a 10-point Likert-type scale, indicated that 23.4% of the sample perceived that HPV infection could cause serious disease, with an overall average value of 7.7, and only 7.5% believed that they were at high risk of contracting HPV. Regarding the belief that the HPV vaccination was effective in preventing cancer, the reported mean value was 8.7, and the importance of vaccinating both women and men against HPV was perceived by 73.7% of participants.

Almost three-quarters (73.2%) of the respondents were aware that the HPV vaccination was recommended. The results of the multivariate logistic regression model regarding the effects of the different factors on the likelihood that participants had this knowledge indicated that women (OR  =  2.24; 95% CI  =  1.13–4.41), HCWs or students in health sciences (OR  =  1.97; 95% CI  =  1.03–3.75), and those who had acquired information regarding HPV vaccination from physicians (OR  =  3.4; 95% CI  =  1.04–11.11) were more likely to be aware that the HPV vaccination was recommended for them (Model 1 in Table 2).

The most frequently cited reasons for vaccinating were self-protection from infection (77.6%) and from cervical/oral/penile/anal cancer (68.9%), followed by knowing that the vaccination was offered free of charge (46.2%), awareness of the severity of HPV disease (43%), to protect their partner from the infection (42.6%), and perception of being at risk of infection (24.2%). The multivariate logistic regression model revealed that HCWs or students in health sciences (OR  =  2.84; 95% CI  =  1.29–6.28), those who believed that HPV infection could cause serious disease (OR  =  1.25; 95% CI  =  1.01–1.56), and those who had a higher number of oral sexual partners in the last year (OR  =  1.49; 95% CI  =  1.17–1.89) were more likely to have received the HPV vaccination because they perceived themselves at risk of infection (Model 2 in Table 2). Having received the HPV vaccination because they believed that it was effective in preventing cancer was indicated by 68.9% of the participants, and this was more likely to be reported by females (OR  =  3.14; 95% CI  = 1.58–6.23) and Italians (OR  =  3.91; 95% CI  =  1.65–9.26) (Model 3 in Table 2).

Almost all participants (99.6%) had received information about the HPV vaccination. The most reported source was the university (78.3%), followed by the Internet (32.5%) and friends/colleagues/relatives (26.5%). Only 16% reported that they acquired information from physicians. Moreover, 9.8% were interested in acquiring more information.

## 4. Discussion

This cross-sectional survey offers several valuable insights for public health authorities into the reasons for having received HPV vaccination among an eligible adult population in Italy and the factors influencing it.

When assessing the different reasons for receiving the vaccination, the findings revealed that the participants cited protecting themselves from HPV infection and from the associated diseases as primary reasons. These results are in line with previous research conducted in other countries among different groups of individuals regarding different vaccinations [7,11,12,13,14]. However, the finding that only 11.2% had been vaccinated because their physician recommended it is very surprising. This lack of recommendation is disturbing since it is widely recognized that HCWs play an essential role in influencing public acceptance of vaccines, and previous surveys found that their advice positively predicted vaccination intention with higher compliance [15,16,17,18,19]. Health policy strategies are needed, and closer collaboration between medical professionals is mandatory, mainly primary healthcare physicians, public health professionals, and gynecologists, to provide essential, consistent, and accurate information to parents and to their patients during routine visits, because recommending HPV vaccination is within the scope of their working activity. However, it is necessary to underline that, although almost all participants had received information about the HPV vaccination, less than one-fifth used HCWs as a source and the Internet was one of the most used sources, in line with previous findings [20,21,22,23]. Moreover, the key role of HCWs in helping their patients to have adequate knowledge and favorable attitudes is confirmed by the results that those who had acquired information regarding HPV vaccination from physicians were more likely to be aware that this vaccination was recommended for them. By contrast, more than one-quarter of the respondents had never received a recommendation by their physician and they did not know that the vaccination was offered free of charge for them. These results are of particular concern and underline the pivotal role of effective information dissemination, emphasizing the urgent necessity of improving communication strategies to ensure that individuals are informed about the vaccination, its benefits, and how to access it. This need is also due to the additional concern that the Internet was one of the main sources of information, and the abundance of negative or unreliable information related to vaccines propagated through this source is well documented and has also been found to be associated with unwillingness or hesitancy [24,25,26,27,28].

With regard to the attitudes towards HPV infection, it is noteworthy that less than one-quarter of the sample considered that it could cause serious disease. Another finding was that the mean value of the perceived personal risk of being infected by HPV was 5.6, with only 7.7% expressing high concern. These findings are in line with data from previous studies regarding the willingness or hesitancy to receive HPV vaccination and highlight a potential gap in understanding the severity and personal susceptibility to HPV infection [29,30,31]. Therefore, interventions that address these perceptions through targeted education may be important in promoting preventive behaviors, empowering individuals to make well-informed decisions regarding their health and well-being.

Evaluating the results of the multivariate logistic regression analysis of the influencing factors of the different outcomes of interest, it is important to underline that among all socio-demographic characteristics, gender and nationality were found to have a significant impact. Specifically, females were more likely to be aware that the HPV vaccination was recommended for them and to have received the vaccination because they perceived themselves as being at risk of being infected. This finding agrees with several previous studies conducted in different countries showing that females generally have a higher level of knowledge and recognize the importance of immunization against HPV than males [32,33,34,35,36]. The reason for this gender gap may be related to the health promotion efforts to prevent HPV infection that have primarily been centered on cervical cancer and, therefore, females may be more likely to be aware of the potential impact of this infection on them and of the benefits of the vaccination, with consequent better health-seeking behavior compared to men. Interestingly, Italian participants were more likely to have received the vaccination to protect themselves from HPV-related diseases. This may be related to the lack of adequate information regarding the risk associated with HPV infection, the importance of this vaccination, and also to the difficulty in accessing vaccination services in their country of origin. Another aspect to note is that, as expected, HCWs and students in health sciences were more likely to be vaccinated because they were aware that it was recommended for them and because they felt they were more at risk of acquiring the infection than other groups. This finding has been previously reported in several surveys conducted in this country and other countries regarding knowledge, attitudes, and behavior regarding different vaccinations [9,37,38,39,40,41], suggesting that medical education plays a crucial role in determining better knowledge, confidence, and behaviors regarding vaccines in general. This is also important since higher confidence in vaccines is a prerequisite for HCWs’ attitudes and behaviors and for vaccination decision-making for themselves and for their recommendations to patients. This higher confidence may be explained by the fact that they are more informed about infectious diseases and their responsibility to provide preventative counseling, either in the present or in the future for students who will become HCWs, than those from other fields. Thus, efforts should be made to ensure more frequent contact with patients. Finally, it was not surprising that respondents with a higher number of oral sexual partners in the last year were more likely to have received the vaccination because they believed themselves to be at risk of HPV infection. This suggests a relationship between individuals’ sexual behaviors and their perception of susceptibility to HPV infection, emphasizing the need for targeted interventions, especially among those belonging to high-risk groups.

The results of this survey should be considered in light of certain potential methodological limitations. Firstly, this was a cross-sectional survey and, therefore, causal relationships between the independent variables and the outcomes of interest could not be determined. Secondly, using a sample from one vaccination center, mainly represented by university’s students, may constrain the representativeness of the subjects enrolled and, subsequently, the generalizability of the findings to the whole population of Italian adults eligible for HPV vaccination. Thirdly, all information collected was self-reported, and caution should be exercised when interpreting sensitive questions, for example, those regarding sexual behaviors, because we cannot rule out social desirability bias due to not responding truthfully. However, this potential bias has been partially minimized because the participants were given assurance that their answers would be anonymous and confidential and the questionnaire was self-administered. This approach aimed to encourage honest responses.

In conclusion, the implication of these results is important and indicates the need for multidisciplinary efforts to effectively develop targeted educational programs and health interventions with tailored messaging, not only to improve the level of knowledge about HPV but also to foster positive attitudes toward the vaccination to increase its coverage for the prevention of HPV-associated diseases.

## Figures and Tables

**Table 1:** Main socio-demographic and general characteristics of the sample.

Characteristics	*N*	%
Age, in years	26.6 ± 7.3 (19–65) *	
Gender		
Male	140	50
Female	140	50
Sexual orientation		
Heterosexual	183	70.4
Bisexual/homosexual/pansexual	77	29.6
Marital status		
Married/cohabiting	35	12.8
Unmarried/separated/divorced	238	87.2
Italian nationality		
Yes	236	83.7
No	46	16.3
HCWs/students in health sciences		
Yes	160	56.9
No	121	43.1
Having at least one HCW parent		
Yes	63	22.3
No	219	77.7
Having at least one chronic medical disease		
Yes	38	13.7
No	239	86.3
Having ever had an HPV screening test		
No	220	78.6
Yes	60	21.4
Having tested positive	31	51.7
Having tested negative	29	48.3
Having ever had complete sexual or oral intercourse experince		
Yes	234	84.5
No	43	15.5
Age, in years, at first complete sexual intercourse experience	18.3 ± 3 (13–30) *	
Age, in years, at first oral intercourse experience	17.6 ± 3 (12–30) *	
Total number of complete sexual intercourse partners	4 ± 6 (0–50) *	
Total number of oral sexual partners	4.7 ± 8.9 (0–100) *	
Number of complete sexual intercourse partners in the last year	1.2 ± 1.3 (0–10) *	
Number of oral sexual partners in the last year	1.2 ± 2 (0–20) *	
Condom use during sexual intercourse		
Never	24	11.2
Rarely	19	8.8
Sometimes	39	18.1
Often	52	24.2
Always	81	37.7

Number for each item may not add up to the total number of the study population due to missing values. * Mean ± standard deviation (range).

**Table 2:** Multiple logistic regression analysis of associated factors with the outcomes of interest.

Variable	OR and 95% CI	*p* Value
Model 1. Awareness that HPV vaccination was recommended for them before being informed by the vaccination center Log likelihood = −118.67, χ^2^ = 24.31 (5 df), *p* = 0.0002		
Females	2.24 (1.13–4.41)	0.02
HCWs or students in health sciences	1.97 (1.03–3.75)	0.04
Having acquired information regarding HPV vaccination from physicians	3.4 (1.04–11.11)	0.042
Younger	0.95 (0.92–1.01)	0.051
No need for additional information regarding HPV vaccination	0.38 (0.15–1.01)	0.051
Model 2. Having received the HPV vaccination because they perceived themselves at risk of being infected Log likelihood = −97.57, χ^2^ = 33.74 (6 df), *p* < 0.0001		
Higher number of oral sexual partners in the last year	1.49 (1.17–1.89)	0.001
HCWs or students in health sciences	2.84 (1.29–6.28)	0.01
Believing that HPV infection could cause serious disease	1.26 (1.01–1.56)	0.037
Need for additional information regarding HPV vaccination	2.31 (0.73–7.31)	0.153
Males	0.63 (0.29–1.36)	0.238
Bisexual/homosexual/pansexual	0.64 (0.29–1.41)	0.265
Model 3. Having received the HPV vaccination because they believed that it was effective in preventing cancer Log likelihood = −123.22, χ^2^ = 35.58 (6 df), *p* < 0.0001		
Females	3.14 (1.58–6.23)	0.001
Italian nationality	3.91 (1.65–9.26)	0.002
Unmarried/separated/divorced	2.36 (0.98–5.71)	0.056
Being aware that HPV vaccination was recommended for them before being informed by the vaccination center	1.76 (0.91–3.46)	0.098
Believing that HPV vaccination was effective in preventing cancer	1.16 (0.93–1.44)	0.179
At least one chronic medical disease	1.61 (0.62–4.17)	0.324

## Data Availability

The anonymous data presented in this survey are available on request from the corresponding author.
